# Reactive Thrombocytosis: A Bizarre Consequence of Splenectomy

**DOI:** 10.7759/cureus.57455

**Published:** 2024-04-02

**Authors:** Harshita J, Sourya Acharya, Shreyash Huse, Ankita Sachdev

**Affiliations:** 1 Department of Medicine, Jawaharlal Nehru Medical College, Datta Meghe Institute of Higher Education & Research, Wardha, IND

**Keywords:** thrombotic events, budd chiari syndrome, portal hypertension, bone marrow, platelets

## Abstract

Platelets are blood components produced in the bone marrow and are essential in forming blood clots. Thrombocytosis refers to a condition that causes the excess production of platelets in the body. When it develops as a reaction to an infection, trauma, or surgery, it is known as secondary or reactive thrombocytosis. Although thrombocytosis is typically a self-limiting disorder, it can frequently result in hemorrhagic or thrombotic events. Extreme thrombocytosis may trigger thrombotic events. Therefore, clinicians must be aware of the complications of thrombocytosis. In this case report, a 35-year-old female, known to have portal hypertension and Budd-Chiari syndrome, presented with complaints of weakness and tingling in her hands persisting for eight days. She disclosed that she had undergone an elective splenectomy as part of her management for portal hypertension and Budd-Chiari syndrome eight days prior.

## Introduction

The platelet is a small, anucleated cell derived from the hematopoietic lineage via the megakaryocyte. Through adhesion, activation, and aggregation, which are initiated by tissue injury, platelets contribute to hemostasis by stimulating coagulation factors and other mediators [1]. Furthermore, these series of actions constitute the essential biological mechanisms for multiple phases of wound healing. By efficiently adhering to the vascular endothelium, aggregating with other platelets, and starting the coagulation cascade, the platelets play a role in maintaining hemostasis [1]. This way, they prevent significant blood loss by creating a fibrin mesh. Thrombocytosis is when the platelet count exceeds the average value of 450,000/μl [2]. Thrombotic occurrences are a hallmark of the hypercoagulable state, which can result from either hereditary or acquired disorders. Life-threatening thrombotic events can be brought on by extreme thrombocytosis [2]. Medical professionals must understand the consequences linked to thrombocytosis. Reactive thrombocytosis following splenectomy occurs in approximately 78% of cases. It is widely known that thrombosis, which has a 5% incidence, is associated with an increased platelet count following splenectomy [2].

Primary thrombocytosis is the disorder of excessive, unregulated production of platelets by the progenitor cells of the bone marrow, which is quite different from reactive thrombocytosis. Reactive thrombocytosis is caused by a physiological megakaryocyte reaction to raised levels of thrombopoietin and inflammatory cytokines in the blood, which, in turn, causes an increase in the megakaryocyte synthesis of platelets [3].

## Case presentation

**Figure 1:**
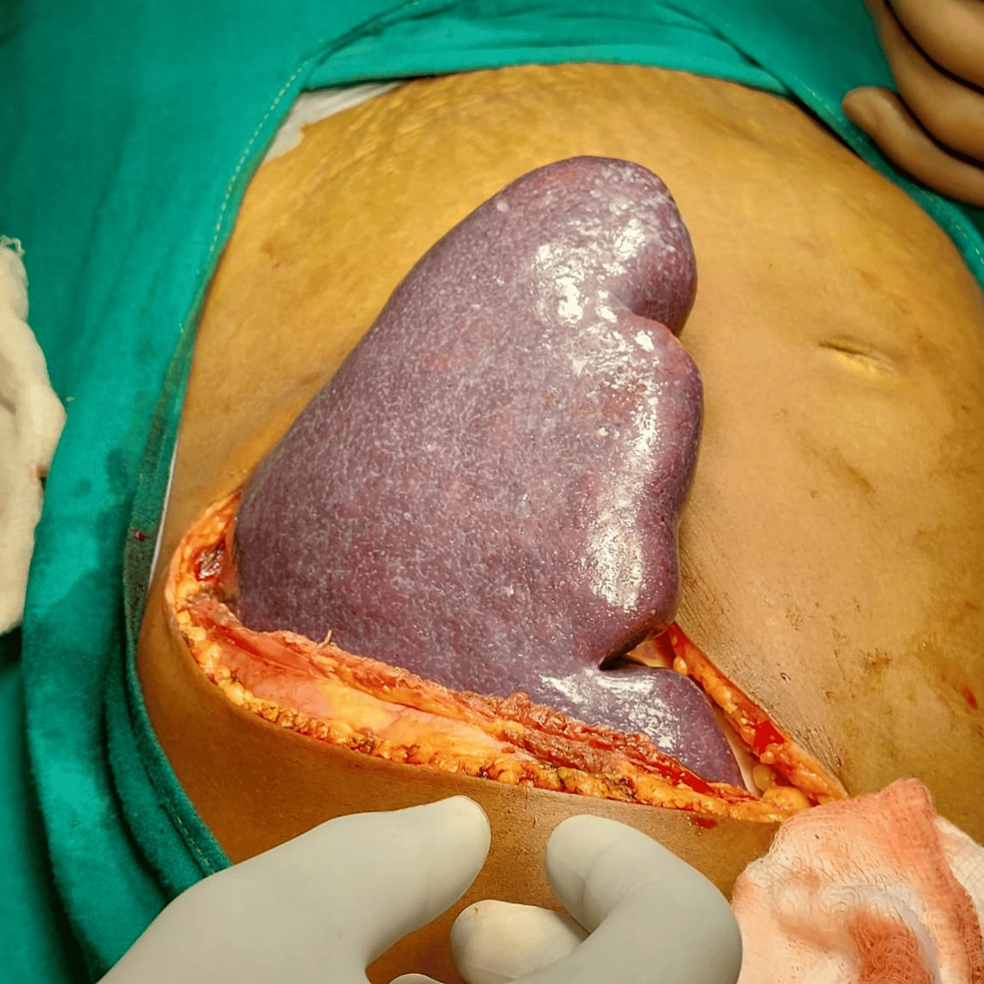
Intraoperative image showing splenomegaly The image was taken by the author.

A 35-year-old non-diabetic female, a known case of portal hypertension with Budd-Chiari syndrome who had been managed with an elective splenectomy, presented to the outpatient clinic with the chief complaints of weakness, dizziness, and tingling in her hands eight days after the splenectomy. An intraoperative image of the patient’s splenectomy is shown in Figure 1. On examination, the patient was afebrile. The pulse was regular, with a rate of 80 beats per minute. The peripheral pulsations were palpable. Her blood pressure was 110/70 mmHg, and her respiratory rate was 18 breaths per minute. Icterus and pallor were absent. There were no petechial rashes on the body. The cardiovascular, respiratory, and central nervous system examinations were within normal limits. A local examination revealed a healthy suture site, ruling out a surgical site infection. Blood investigations of the patient as compared to the normal reference range are shown in Table 1.

**Table 1:** Blood investigations of the patient

	Blood investigations	Normal range (female)	Patient’s value
1	Total leucocyte count	4,000-11,000/mL	18,600/mL
2	Absolute platelet count	1.5-4 lakh/microliter	6.2 lakh/microliter
3	Hemoglobin	12-16%	7.90%
4	Hematocrit	36-46%	24.60%
5	Serum alkaline phosphatase	30-100 IU/L	142 IU/L
6	Aspartate aminotransferase	9-25 IU/L	29 IU/L
7	Alanine aminotransferase	7-30 IU/L	21 IU/L
8	Prothrombin time	11-14 seconds	13.2 seconds
9	Activated partial thromboplastin time	20-40 seconds	30.8 seconds
10	Erythrocyte sedimentation rate	0-15 mm/hour	40 mm in the first hour
11	C-reactive protein	<1 mg/dL	4.9 mg/dL

**Figure 2:**
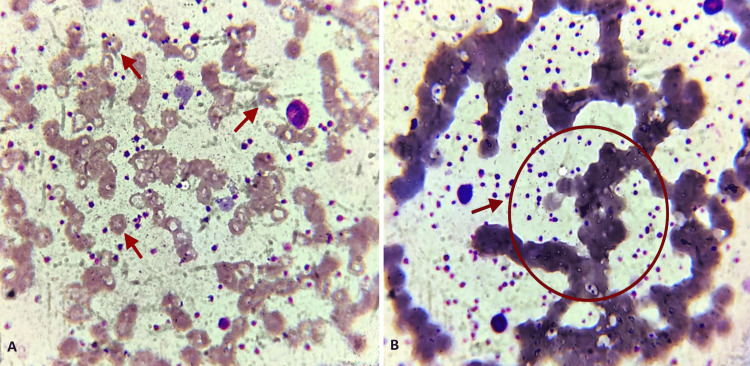
(A) Peripheral smear stained with Leishman stain showing increased platelet count with mild anisopoikilocytosis, a few microcytic hypochromic RBCs, and pencil cells. (B) Peripheral smear stained with Leishman stain (oil immersion view: 100x) showing normocytic normochromic RBCs and platelets appeared in clumps with a few giant platelets The image was taken by the corresponding author.

The peripheral smear showed an increased platelet count in clumps as seen in Figure 2A, with normocytic normochromic RBCs, mild anisopoikilocytosis, and a few microcytic hypochromic RBCs and pencil cells as seen in Figure 2B. Moreover, neutrophilic leukocytosis was present, and no hemoparasites were seen.

The diagnosis of post-splenectomy reactive thrombocytosis was made, and the patient was started on an injection of heparin (2,500 IU twice a day) and a tablet of aspirin 150 mg with adequate hydration to avoid thrombosis. On the third day of therapy, a total blood count investigation was done, which exhibited a platelet count of 714,000/mcL (normal range: 1.5-4 lac/mcL). The patient was advised to continue injections of heparin (2,500 IU twice a day) and aspirin (150 mg). On the second week of follow-up, the platelet count was 6.5 lakh/microliter (normal range: 1.5-4 lakh/microliter). The patient was asymptomatic. On follow-up after one month, the repeat parameters were as mentioned in Table 2. The patient had been asymptomatic for a month, and therefore, aspirin was stopped.

**Table 2:** Blood parameters after one month

	Blood investigations	Normal range	Patient’s value
1	Absolute platelet count	1.5-4 lac/mL	395,000/mcL
2	Erythrocyte sedimentation rate	0-15 mm/hour	30 mm in the first hour
3	C-reactive protein	<1 mg/dL	4.2 mg/dL
4	Total leukocyte count	4,000-11,000/mL	10,570/mL

## Discussion

Thrombocytosis is often an accidental laboratory finding. It is referred to as a platelet count exceeding 450,000/mcL in adults. This abnormality is classified as “reactive thrombocytosis” when the increase in platelets is found to be an external cause [3]. Overproduction of thrombopoietic factors acting on either the megakaryocytes or their precursors is assumed to be the cause of reactive thrombocytosis [4]. Increased levels of such growth factors are seen in a variety of inflammatory, neoplastic, traumatic, and infectious events [5]. Interleukin-6 is the key factor in reactive thrombocytosis out of all the growth factors identified. Since the spleen is the primary location for platelet destruction, it is vital for regulating platelets. As a result, thrombocytosis is frequently associated with hyposplenism. Following splenectomy, reactive thrombocytosis is an anticipated outcome; the platelet count peaks in one to three weeks and returns to normal in the following weeks, months, and, in rare cases, years [6]. Typically, underlying disorders, rather than thrombocytosis, are the major cause of symptoms. Rarely, thrombotic events such as acute myocardial infarction, pulmonary embolism, and mesenteric vein thrombosis can be brought on by extreme thrombocytosis [7].

In patients with increased platelet counts, the underlying cause can either be a primary defect or a reactive cause. Further, the associated immediate risks from thrombocytosis must be evaluated in the patient. Following that, thrombocytosis needs to be controlled to avoid adverse consequences. It is important to treat patients with essential thrombocytosis who have experienced thrombotic episodes and have cardiovascular risk factors [8]. Various pharmacological agents like acetylsalicylic acid, hydroxyurea, anagrelide, interferon alpha, ticlopidine, enoxaparin, and plasmapheresis can be used in the management of extreme thrombocytosis. In cases of essential thrombocytosis, hydroxyurea is considered to be superior to anagrelide in reducing the risks of major hemorrhagic and thrombotic events [8,9]. When using aspirin, patients with thrombocytosis should be cautious about the potential risk of bleeding. Patients using hydroxyurea need to be watched closely for leukemic transformation [10,11]. Since high platelet aggregation is the result of thrombocytosis, regardless of the underlying etiology, the preferred line of treatment is the administration of aspirin, which prevents platelet aggregation [12,13].

## Conclusions

This is a rare case of post-splenectomy reactive thrombocytosis in a patient who had a known case of portal hypertension with Budd-Chiari syndrome. Although reactive thrombocytosis is benign, its underlying causes, such as cancer, connective tissue abnormalities, or chronic infections, may carry a higher risk of unfavorable outcomes. So, treating the underlying cause of thrombocytosis is crucial in reactive thrombocytosis. Since it has an impact on diagnosis, prognosis, and treatment, the distinction between primary and secondary thrombocytosis is crucial.
